# Challenges in the evaluation of suicide prevention measures and quality of suicide data in Germany, Austria, and Switzerland: findings from qualitative expert interviews

**DOI:** 10.1186/s12889-024-19726-w

**Published:** 2024-08-13

**Authors:** Sophia Werdin, Kaspar Wyss

**Affiliations:** 1 Swiss Tropical and Public Health Institute, Allschwil, Switzerland; 2 University of Basel, Basel, Switzerland

**Keywords:** Mental health, Suicide prevention, Evaluation research, Data quality, Epidemiological monitoring, Qualitative research, Stakeholder participation, Europe

## Abstract

**Background:**

Suicide prevention requires diverse, integrated, and evidence-based measures. Comprehensive evaluation of interventions and reliable suicide data are crucial for guiding policy-making and advancing suicide prevention efforts. This study aimed to analyze current issues and gaps in the evaluation of suicide prevention measures and the quality of suicide data in Germany, Austria, and Switzerland to derive specific recommendations for improvement.

**Methods:**

Online, semi-structured interviews were conducted with 36 experts in suicide prevention from Germany, Austria, and Switzerland, covering insights from policy, science, and practice. The interviews took place between September 2022 and February 2023, were audio-recorded, transcribed verbatim, and analyzed using the Framework method.

**Results:**

While solid evidence supports the effectiveness of some suicide prevention interventions, experts indicated that the evaluation of many other measures is weak. Conducting effectiveness studies in suicide prevention presents a range of methodological and practical challenges, including recruitment difficulties, choosing adequate outcome criteria, ethical considerations, and trade-offs in allocating resources to evaluation efforts. Many interviewees rated the quality of national suicide statistics in Germany, Austria, and Switzerland as comparatively high. However, they noted limitations in the scope, timeliness, and reliability of these data, prompting some regions to implement their own suicide monitoring systems. None of the three countries has national routine data on suicide attempts.

**Conclusion:**

While some challenges in evaluating suicide prevention measures are inevitable, others can potentially be mitigated. Evaluations could be enhanced by combining traditional and innovative research designs, including intermediate outcomes and factors concerning the implementation process, and employing participatory and transdisciplinary research to engage different stakeholders. Reliable suicide data are essential for identifying trends, supporting research, and designing targeted prevention measures. To improve the quality of suicide data, a standardized monitoring approach, including uniform definitions, trained professionals, and cross-sector agreement on leadership and financing, should be pursued. This study provides actionable recommendations and highlights existing good practice approaches, thereby supporting decision-makers and providing guidance for advancing suicide prevention on a broader scale.

**Supplementary Information:**

The online version contains supplementary material available at 10.1186/s12889-024-19726-w.

## Background

Suicidality is a major public health concern, leading to more than 700,000 deaths worldwide every year [1] and impacting many more individuals who attempt suicide and experience suicidal ideation [2]. Although the majority of suicides occur in low- and middle-income countries, age-standardized suicide rates peak in high-income countries [1]. Despite various efforts to prevent suicidal behavior and the advantages of high living standards and advanced health systems, Germany, Austria, and Switzerland report suicide rates above the average of the European Union [3]. While suicide is commonly linked to mental illness [4], complex and multifaceted risk factors make it a highly heterogeneous phenomenon [5, 6]. A holistic approach to suicide prevention (SP) that integrates diverse measures across all prevention domains is necessary [7, 8]. Population-based SP strategies include, for example, restricting access to means of suicide and implementing media guidelines for responsible suicide reporting [9]. High-risk SP strategies focus on targeted interventions for individuals at suicide risk, including specific treatments for patients in psychiatric hospitals [9]. In this paper, we do not address assisted suicides, as they typically have different underlying causes and require distinct prevention strategies [10].

In public health and healthcare, decisions should be guided by the best available evidence and take into account practical aspects such as feasibility of implementation, sustainability, and acceptability [11]. Scientific evidence comprises research findings, with evidence quality categorized into high, moderate, low, and very low, depending on study design and factors such as study limitations, consistency, and data precision [12]. High-quality evidence comes from methodically sound studies that minimize systematic errors through their design and execution, such as double-blind randomized controlled trials [12]. In contrast, low-quality evidence stems from studies with methodological flaws, such as inadequate control of confounding factors, small sample sizes, or bias, which limit the credibility and generalizability of their findings [13]. Comprehensive evaluations form the foundation for assessing the implementation and effectiveness of SP measures, providing information that guides the setting of priorities, the allocation of funds, and the development and refinement of SP strategies. Research findings enhance our understanding of suicidality and its preventability, and help identify gaps in the SP landscape. Insufficient evidence on intervention outcomes is likely to pose significant challenges in securing continuous funding and support from stakeholders, potentially jeopardizing their sustainability.

Since the primary aim of SP measures is to prevent suicides and suicide attempts, having access to reliable and valid suicide data is pivotal for their evaluation. The validity of epidemiological and sociodemographic theories about suicidality hinges on the reliability of suicide statistics, which, however, has been addressed in only a few scientific studies covering a limited number of countries [14]. The World Health Organization considers the availability and quality of suicide data globally to be poor, pointing to issues of underreporting and misclassification [15]. In particular, indications of the frequency of suicide attempts were described as being only rough estimates, as few countries have a system in place for suicide attempt monitoring [16]. Since a suicide attempt is a major predictor of death by suicide [15], long-term monitoring of suicide attempts and their characteristics is crucial for informing and guiding SP efforts [16].

In this paper, we use the term *suicides* for deaths by suicide. *Suicide attempts* refer to instances where an individual has attempted to take his or her own life but did not result in death. Correspondingly, we use the term *suicide statistics* in relation to deaths by suicide and *statistics on suicide attempts* in relation to suicide attempts. *Suicide data* encompasses all data related to both deaths by suicide and suicide attempts.

Given the significant burden of suicidality on individuals, families, communities, and society at large, it is important to understand the effectiveness of SP interventions, the mechanisms used for their evaluation, and the quality of suicide data that guide prevention efforts. This study aimed to explore current issues and gaps in SP evaluation and suicide data, while capturing recent advancements. Drawing on insights from experts in Germany, Austria, and Switzerland, we aim to formulate specific and actionable recommendations to enhance both evaluation efforts and the quality of suicide data.

## Methods

The detailed research methods employed in this study have previously been described [17]. Here, we summarize key aspects of the methodological approach. Reporting in this manuscript was guided by the Consolidated Criteria for Reporting Qualitative Research: 32-item checklist [18].

### Study design and setting

We conducted a qualitative study to explore the SP landscape in Germany, Austria, and Switzerland, three neighboring, high-income countries in Central Europe with comparable socio-cultural, economic, and political-organizational characteristics. From September 2022 to February 2023, we held one-on-one online interviews with SP experts from these countries [17].

### Study participants and recruitment

36 SP experts participated in our study, designated as experts due to their specialized knowledge, relevant experience, and roles as informants [19]. We focused on expert knowledge derived from professional involvement in SP. Individuals were classified as experts if their professional responsibilities significantly involved activities such as planning, coordinating, implementing, or evaluating SP measures. Potential interviewees were selected using purposive sampling. The process of identifying and choosing experts relied on the researchers’ judgement. The first author (SW) reviewed potential participants from pertinent SP organizations, scientific publications, programs, and other initiatives contributing to SP. This strategy and the selection of individuals were discussed with the co-author (KW) and other colleagues involved in SP research. Potential interviewees were contacted via email. A total of 68 SP experts were approached; 16 did not respond, and 16 declined participation, primarily due to time constraints. No expert declined after agreeing to participate in our study [17].

The participating experts were evenly distributed across Germany, Austria, and Switzerland, with 12 individuals from each country. Furthermore, 12 experts each had a primary professional background in policy, science, and practice. The sample size of 36 individuals was strategically chosen to ensure a balanced geographical representation and a comprehensive range of diverse perspectives across the field of SP. This diversity was considered essential for obtaining a holistic understanding of the current challenges and opportunities in these countries, thereby enhancing the applicability of our findings. With this sample size, we anticipated being able to comprehensively explore our research interests and achieve data saturation - the point at which additional data collection no longer yields new insights [20].

Policy experts included, for example, employees of federal health agencies and members of SP societies and associations. Scientific experts comprised researchers at universities or university hospitals specializing in suicide research. Practitioners, such as psychotherapists, psychiatrists, employees from SP projects and counseling services, and other professionals who regularly interact with individuals at suicide risk, were also interviewed [17]. Many experts in the field of SP engage in activities beyond their primary professional roles, assuming multiple responsibilities across the broader SP landscape. For example, several interviewees categorized as scientific experts also provide clinical care or engage in policy-making alongside their research activities. Similarly, some experts categorized within the policy domain actively contribute to scientific research. The classification of interviewees into professional domains is not intended to impose rigid segmentation, but rather to ensure that diverse perspectives are adequately represented [17].

Participation was voluntary. Participants received detailed study information via postal mail prior to the interview. Written, informed consent was obtained from all interviewees. There was no compensation for study participation.

### Data collection

Data collection involved online, semi-structured interviews via Zoom, guided by an interview protocol with open-ended questions (see Additional file 1). The interview guide was developed and refined by a research team comprising public health experts experienced in evaluating SP projects. The instrument underwent a pretest to assess its logic, comprehensibility, and completeness during face-to-face interviews with two colleagues involved in SP research. After initial interviews with the study participants, we made slight adjustments to the wording of some questions and reduced the length of the instrument to improve clarity and decrease the time required. The interview guide covered the following topics: (1) aspects on the national SP approach, (2) the evaluation and (3) effectiveness of SP measures, (4) the availability and quality of suicide data and evidence in SP, (5) challenges in SP, (6) the impact of the Coronavirus disease 2019 (COVID-19) pandemic, and (7) best practices and optimization potentials. The focus on specific interview topics corresponded with the primary professional perspectives of the experts. Participants were emailed the interview guide one week prior to their interviews [17].

The first author (SW, female, PhD candidate at the Swiss Tropical and Public Health Institute, trained and experienced in qualitative research methods) conducted the interviews primarily in German, except for one in English. The interviews were audio-recorded, transcribed verbatim, and anonymized following the basic transcription system of Dresing and Pehl [21]. Upon request, the participants received the transcript of their interview by email.

### Data analysis

The data were analyzed using the Framework method [22], organizing qualitative data into thematic codes that were deductively derived from the interview guide. A coding tree, including the main themes and associated sub-themes, was built based on the interview data and used to systematically apply the codes to the relevant text segments. Data reduction and analysis were conducted using a theme matrix structured as “cases by codes”, facilitating the comparison of data both across different cases and within individual cases [22]. The interviewer (SW) coded and analyzed the data. Data management and analysis were conducted using MAXQDA.

This manuscript presents our findings on the evaluation of SP measures and the quality of suicide data, along with associated challenges, best practices, and areas for enhancement. A previously published, complementary manuscript [17] addresses our findings on the perceived role of SP and national SP strategies in Germany, Austria, and Switzerland, conditions for collaboration in SP, the acceptance of SP measures as well as the impact of the COVID-19 pandemic on SP, along with associated challenges, best practices, and areas for enhancement.

## Results

### Sample characteristics

Our study sample included 15 female (41.7%) and 21 male experts with an average age of 53 years. The duration of the interviews ranged from 22 to 69 min, averaging at 42.5 min. A more detailed description of the study sample as well as the geographic distribution of the participants in Germany, Austria, and Switzerland can be found in our previously published, complementary manuscript [17].

### Challenges in evaluating the effectiveness of suicide prevention measures

Most experts reported that few SP measures have been evaluated based on scientific criteria. Comprehensive and reliable results from evaluations using robust research methods, especially longitudinal studies assessing medium- and long-term effects, were described to be rare in SP. According to the interviewees, the extent and quality of evaluation efforts vary widely, resulting in a lack of robust evidence supporting the effectiveness of many SP initiatives.

For example, concerns were raised about the quality of evidence for a wide range of clinical interventions in suicide risk management. In this context, several experts highlighted a lack of standardization in treating individuals at suicide risk. Moreover, they criticized the reliability of current practices in suicide risk assessment, considering them mere guesswork.

«Surprisingly, for a lot of what we do in [clinical] practice, in daily routine, little data is available. When it comes to the assessment of suicidality in clinical practice, the use of antidepressants in severely depressed, suicidal people - there is really a big leap from evidence to daily clinical practice.» (Participant 18, male, science, Austria).

«And I do that [clinical practice] to the best of my knowledge and belief. However, I [the psychiatric hospital] do not ensure that certain procedures are standardized. I have been able to demonstrate this in my own investigation: every hospital basically does what it wants. Not in a bad sense at all, but to the best of their knowledge and with the conviction that everything what they are doing is good. But [they] do not realize that things could actually work better.» (Participant 13, female, science, Germany).

Furthermore, interviewees mentioned that only a few targeted SP projects, such as *GO-ON* in Austria, the online counseling service *U25* in Germany, and the *Attempted Suicide Short Intervention Program* in Switzerland, have been evaluated based on rigorous, scientific standards. Many other projects and measures are not evaluated at all.

«It is certainly a weakness that many individual measures have not been adequately evaluated. We have maintained this website, which was previously managed by the Swiss Federal Office of Public Health, to showcase practical examples of suicide prevention [projects] in Switzerland. […] Many projects are listed, most of which […] have never been evaluated.» (Participant 9, female, policy, Switzerland).

Conducting adequate, valid, and robust evaluations of SP interventions can present a variety of challenges. Several experts noted difficulties in assessing the effectiveness of initiatives designed to enhance awareness and knowledge among the general population, such as media campaigns or public information events. Measuring shifts in public attitudes or behaviors towards SP and directly attributing any observed changes to these awareness efforts is challenging. Furthermore, interviewees highlighted difficulties in recruiting study participants, including limited access to the target group and low response rates, and methodological issues, such as identifying adequate outcome criteria and the absence of a suitable control group. Additionally, ethical considerations, a potential lack of scientific expertise within the project team, and scarce resources dedicated to evaluation purposes were noted as significant challenges. In online or telephone counseling services, clients often remain anonymous, and data protection regulations may impede evaluative efforts. Furthermore, several experts noted that the evaluation periods are typically too short to fully assess the preventive effects on suicidal behavior.

«The evaluations are not sufficiently resourced, and due to this lack of resources, they cannot encompass medium- or longer-term time horizons. We often evaluate the impact right after [the implementation]. However, in suicide prevention, we aim for medium- and long-term [impacts] actually. Therefore, the time horizon [for these evaluations] is not long enough.» (Participant 24, female, science, Switzerland).

«Many projects received funding from this suicide prevention fund over three years, which is simply too short for prevention in most cases. These brief funding periods make it almost, perhaps not impossible, but certainly more difficult to demonstrate tangible effects. This is because prevention, especially if you think of it on a population-wide scale, inherently requires more time.» (Participant 27, female, practice, Germany).

In the case of complex, multi-causal phenomena like suicidality, many experts noted the difficulty of causally linking a positive effect to a specific intervention. Furthermore, within the often geographically limited scope of a prevention measure, suicide rates are usually too low to observe significant changes.

«Of course, we also had long discussions about which indicators we should focus on [in the evaluation]. The suicide rate alone is not appropriate. Because sometimes, especially in times like these [societal crises], simply not observing an increase in the suicide rate is a success. At the same time, suicide must always be seen as a multifactorial event. And this [evaluation based on the suicide rate] becomes particularly challenging when a war breaks out simultaneously with the energy and climate crisis.» (Participant 5, male, policy, Austria).

Due to these inherent limitations, some experts recommended including softer, intermediate outcomes and factors related to the implementation process in SP evaluations.

«There is a development to frame the validation of activities for suicide prevention in a broader, scientific context and not just see the reduction of suicide rates as the central adjusting screw by which all activities are to be measured. This is because the impact on suicide rates is extremely multifactorial and has been shown to be very difficult to influence through individual measures. […] A keyword here would be ‘implementation is evaluation’. […] We measure more on the very direct level of the implementation of our activities.» (Participant 25, male, science, Germany).

«In these population-based studies, we also try […] to better integrate the question of implementation. […] We see extreme heterogeneity within the country, but also internationally, regarding how interventions are implemented. And therefore, it is often unclear, when a study comes out: does it depend on the fact that it may not work in one country because it is less accepted there? Or is it due to the implementation itself? There are of course many gaps and we try to address them with more complex designs.» (Participant 17, male, science, Austria).

Beyond the direct challenges of evaluating SP measures, some study participants noted that even with reliable evidence of an intervention’s effectiveness, its implementation in practice may face barriers.

«There is a significant discrepancy because, although we know what is evident and effective, these insights seldom reach or are minimally reflected in practical implementation. […] Even if we identify hotspots, which are prevalent everywhere including Germany, and approach certain decision-makers, they sometimes block us, reject us, put us off, or claim that prevention is not possible in this context. Well-founded evidence supporting this [methods restriction], which is the strongest, is then blocked or nullified with flimsy arguments, such as the need to protect historical monuments.» (Participant 13, female, science, Germany).

### Availability and quality of suicide data

#### Statistics on suicides

Suicide statistics play an important role in evaluating SP measures and studying patterns and trends in suicidality. In Germany, Austria, and Switzerland, national suicide statistics are published annually and include officially recorded suicides. Most experts rated the quality of the national suicide statistics in these three countries as comparatively high. However, numerous interviewees criticized a lack of relevant information, such as details on the suicide method and the exact locations where these incidents occurred, which are crucial for both evaluation purposes and research. Furthermore, almost all experts stressed that there is a considerable delay in the publication of national suicide statistics, indicating delays of up to several years. This delay hampers the ability to respond promptly to emerging trends and conduct research on current developments in suicidality at the national level, underscoring the need for more timely data dissemination.

«It takes far too long for the Federal Statistical Office to publish the corresponding suicide rates and figures. In other words, the connection between the statistical offices in the federal states and the Federal Statistical Office is still far too cumbersome to be able to influence current developments quickly and in a targeted manner. Great Britain, Ireland, and other countries act much faster. As a result, these countries can implement suicide prevention measures on a regional and much smaller scale directly from these figures, for example, addressing suicide clusters in certain areas or among specific social groups. We cannot do this at all yet.» (Participant 25, male, science, Germany).

During the COVID-19 pandemic, the need for timely access to suicide statistics as an indicator of the population’s mental health became particularly evident, as several experts noted. For example, in Austria, a monitoring project was initiated to facilitate quicker internal analysis of suicide data, demonstrating a proactive approach to responding to mental health challenges during such unprecedented times.

«We have developed a monitoring concept that encompasses overall mental health, with suicidality being one aspect. It was politically agreed upon that we receive unvalidated preliminary data from the Austrian Statistical Office, exclusively for the crisis teams of the individual ministries established in response to COVID-19. We receive this preliminary data nine weeks after the end of each quarter. We are not permitted to publish it, so it is only utilized for consultations within the expert committees. This is extremely helpful because it allows us to gauge the situation in the first half of the year and react much more quickly.» (Participant 5, male, policy, Austria).

Beyond the data captured in official suicide statistics, many experts pointed to a difficult-to-quantify yet potentially high number of suicides that go unreported. As one of several underlying reasons, some interviewees mentioned that during post-mortem examinations, physicians may intentionally avoid labeling a death as suicide to prevent stigmatizing both the deceased and their relatives. Similarly, a reluctance to record suicide attempts was noted.

«Through our discussions, we discovered that general practitioners were often reluctant to code this diagnosis [suicide]. […] This choice was made to avoid stigmatizing patients in case someone else reads the file. As a result, many cases were simply not recorded.» (Participant 4, male, policy, Germany).

«We have encountered general practitioners who have said: *No*,* I will not harm the family by labeling it ‘suicide’. I write ‘multiple organ failure’ instead.* Consequently, the official statistics are not accurate.» (Participant 30, female, practice, Austria).

Suicide methods that are difficult to trace, such as poisoning, may lead to deaths being mistakenly recorded as natural deaths or accidents. In this context, one researcher noted that the autopsy rate in a country appears to impact the official suicide rate, with fewer autopsies correlating with fewer deaths identified as suicides. Additionally, several experts highlighted that the methods used to document suicides and the criteria for defining a suicide or suicide attempt vary among different agencies, including the police, healthcare facilities, and emergency medical services.

«We thoroughly examined the question: How does Statistics Austria determine that this [death] was a suicide? And [we found out that] the police or the primary care physician take a completely different approach.» (Participant 30, female, practice, Austria).

«When someone attempts suicide but survives, and the rescue team arrives to resuscitate him, he is then taken to the hospital. Suppose he is treated in the intensive care unit for three weeks with the support of a heart-lung machine but ultimately passes away. In that case, this incident is not recorded as a suicide. In my opinion, the initial cause was the suicide attempt, as he intended to take his own life. I believe such cases should be included in the [suicide] statistics, but they are not.» (Participant 13, female, science, Germany).

To mitigate this issue, several experts emphasized the critical need to establish uniform standards for documenting suicides and suicide attempts, and to ensure that physicians and other professionals involved in recording receive adequate training.

«To guarantee quality, it is essential to provide training for doctors. They must be trained to ensure that every admission is evaluated for this [potential suicide attempt and ideation]. In fact, asking about suicidal behavior in the initial anamnesis is a standard practice in every [psychiatric] hospital. This includes inquiries whether suicidal behavior is present in the patient’s own life, or whether the mother, father, or second-degree relatives have attempted or committed suicide. Although this is standard practice, it is often omitted.» (Participant 4, male, policy, Germany).

Additionally, strict data protection regulations were identified as a potential barrier that could compromise the validity of suicide statistics.

«Regarding suicides - and this does not seem to bother anyone but me - I put a big question mark behind the figures [official statistics] since the introduction of the General Data Protection Regulation. Since we have that, some suicides are no longer classified under the ‘X’ category, according to the ICD-10 classification, but are instead recorded under the ‘R’ category for other causes of death. This aims to protect the individual, given that this data could theoretically be accessed by the public. […] But are we talking about ten suicides or are we talking about 20, 30, hundreds of suicides? When you look at the trend for ‘R’ diagnoses, which is also publicly available - it goes UP! And it increases steeply! Now, I do NOT know: are these genuinely other causes of death? What did these people die of? Or are these actually all suicides?» (Participant 13, female, science, Germany).

Due to the limitations of national suicide statistics, experts described that some federal states and cantons have initiated their own suicide monitoring systems, which offer more comprehensive and current data. For example, the federal state Carinthia in Austria took a significant step in 2018 by implementing regional, up-to-date suicide statistics that provide extensive background information on each incident. According to several Austrian experts, the Carinthian system is being recognized as a best practice model in Austria, inspiring other federal states to initiate similar suicide monitoring systems.

«Since 2018, we have been compiling up-to-date suicide statistics. […] they are much more precise than the official Austrian suicide statistics. One key advantage of our data is its daily updates. That means we do not have to wait half a year or a year for the official statistics. Moreover, we have many additional parameters that allow us to respond very quickly to emerging trends.» (Participant 20, male, science, Austria).

The Carinthian coordination office for SP gathers the data from multiple sources.

«We obtain data from the operational protocols of the executive force, which we then cross-reference with the intervention protocols from the Red Cross’s crisis intervention team and keep up to date. Additionally, we compare this data with records from the psychiatric departments, covering both post-discharge and inpatient suicides. Furthermore, we match the data with the data from psychiatric emergency and crisis services.» (Participant 20, male, science, Austria).

German experts mentioned the establishment of a database for recording suicides and suicide attempts in hospitals, with reporting encouraged on a voluntary basis.

«We have developed a German-language-wide database for suicides in clinics. […] Where we voluntarily, of course - it is not a law - ask all clinics to report these events to us. Everything is anonymous, all data protection regulations are observed. But where you actually get solid data to look at: what is actually happening?» (Participant 13, female, science, Germany).

Furthermore, there are regional efforts in all three countries aimed at conducting more thorough investigations into suicide incidents. For instance, Swiss experts pointed to a collaborative initiative with the cantonal police and public prosecutors in Zurich, which involves cross-referencing records with mortality data.

«In Zurich, based on the police crime statistics, we go to public prosecutors and try to find out more information there. Especially in terms of hotspots, methods, and such things. And we try to be as timely as possible with that.» (Participant 11, female, policy, Switzerland).

#### Statistics on suicide attempts

There is no national monitoring of suicide attempts in Germany, Austria, and Switzerland. Many experts pointed out that defining, recognizing, and consistently recording suicide attempts presents significant challenges, even greater than those associated with recording suicides. One of several underlying reasons is that not all individuals who attempt suicide come into contact with the healthcare system or other state authorities capable of documenting the attempt. Furthermore, distinguishing between a suicide attempt, non-suicidal self-injury, or accidental harm can be difficult, complicating efforts to accurately categorize such incidents.

Several experts mentioned scattered projects and scientific studies that aim to systematically document data on suicide attempts. However, they also noted that these initiatives are usually localized, focusing on specific geographic regions or institutions, especially psychiatric hospitals. As a result, these projects often involve rather small samples or represent narrow target groups, making extrapolation of their suicide attempt data to a broader population less reliable.

«In Austria, there is no central database or collection of suicide attempts and self-harm. Individual projects are being conducted, typically in collaboration with individual hospitals, where the suicide attempts are then recorded. These are all managed through separate ethics applications and research projects. However, obtaining a comprehensive overview in this area is fundamentally challenging.» (Participant 17, male, science, Austria).

Given the importance of reliable suicide data for evaluating SP measures and researching suicidality, numerous experts advocated for a centralized, nationwide monitoring system to record suicides and suicide attempts.

«A suicide registry - This is the biggest gap. We need a Switzerland-wide suicide registry, which is obligatorily anchored in law, like a cancer registry. This would enable us to collect naturalistic data on suicide attempt frequency. This is almost more important than deaths by suicide - a suicide attempt registry. Currently, we are COMPLETELY in the dark. We have NO idea. The only way to remedy this is with reliable data. It must be naturalistic, simple, and anonymized, centralized, so that nobody is afraid of what happens to their data.» (Participant 36, male, practice, Switzerland).

## Discussion

A main finding of our study is that experts in Germany, Austria, and Switzerland shared similar experiences and opinions regarding the evaluation of SP interventions and the availability and quality of suicide data. In the following, we address challenges and gaps in evaluating the effectiveness of SP measures, discuss the role of reliable suicide data, and explore related opportunities for improvement.

### Challenges and opportunities for improvement in the evaluation of suicide prevention measures

Participating SP experts indicated that many SP measures lack comprehensive and reliable evaluation, limiting the evidence supporting their effectiveness. This observation aligns with the findings of numerous studies that have raised concerns about the insufficient or low-quality evidence for various SP efforts [5, 8, 23, 24]. The specific challenges in the evaluation of SP measures identified by the interviewees and confirmed by several other studies highlight the complexity of this research field. O’Connor and Portzky [25] consulted 32 experts from 12 countries and identified the limited reliability of suicide data as the primary challenge in suicide research. The multicausality of suicides and their relatively low incidence in the often geographically limited scope of an SP measure [7, 26] may reduce their suitability as the sole outcome measure [25]. A considerable delay in the publication of national suicide statistics impairs their usability for evaluating broad SP measures [27]. The complexity of SP interventions, particularly those involving multiple components and different sectors and prevention levels, further complicates their evaluation [28]. Beyond mere questions of effectiveness, aspects such as feasibility of implementation, appropriateness, acceptability, cost-effectiveness, transferability, and scalability are crucial but often overlooked [28].

While some challenges in suicide research, such as the impact of external factors on suicidality and the relatively low suicide rates in geographically limited study settings, are inherently inevitable, others could potentially be mitigated. Hereafter, we discuss strategies aimed at facilitating the selection of appropriate outcome measures and the application of feasible research designs in SP evaluations.

Given the difficulties in establishing a causal link between SP interventions and suicide outcomes, coupled with concerns about the quality of suicide data, several interviewees recommended including softer, intermediate outcomes and factors related to the implementation process in the evaluation of SP measures. Intermediate outcomes can serve as proximal effect indicators and are directly linked to an intervention’s objectives and content [29]. According to the World Health Organization, intermediate outcomes that are influenced by SP efforts in the short term can provide indications of the intervention’s long-term impact on suicidal behavior [30]. Intermediate outcomes in SP include changes in the utilization of support services [30], attitudes towards help-seeking, stigma surrounding depression and/or suicide, and the acquisition of protective behaviors [29]. For example, self-management and self-efficacy could be suitable competences for estimating the proximal effect of SP interventions. Individuals at suicide risk often demonstrate heightened sensitivity to emotional distress and negative social signals, as well as a reduced capacity for problem-solving [31]. High self-management competence can help in the recovery from mental illness [32] and is considered a protective factor against suicidality [33]. Similarly, a high level of self-efficacy can help prevent the transition from suicidal ideation to suicidal behavior by encouraging the use of effective coping strategies [34].

We advocate collaborating with different stakeholders to determine meaningful outcome criteria tailored to individual SP measures, considering factors such as the measure’s scope, the potential number of beneficiaries, and the type of intervention. While the primary focus should remain on reducing suicides and suicide attempts [30], including intermediate outcome criteria can be valuable to strengthen the evidence for the effectiveness of SP measures [29, 35], especially when evaluating smaller-scale initiatives in real-world settings. Evaluating additional aspects such as the feasibility of implementation, appropriateness, acceptability, transferability, and scalability, provides a practical and comprehensive assessment of a complex intervention’s relevance and impact [28].

For research into complex phenomena like suicidality, context-specific, flexible, and innovative methods such as adaptive designs or hybrid effectiveness-implementation trials can be beneficial [28]. Furthermore, innovative data science techniques, including machine learning and predictive analytics, have proven effective in analyzing suicidal thoughts or behaviors, identifying risk factors, and predicting outcomes [36], thus demonstrating their value as tools in SP evaluation [35]. Analytical advancements also facilitate the use of real-world data, defined as data collected during routine clinical care, which offer greater precision and power in generating real-world evidence [26]. Utilizing real-world data helps overcome limitations of traditional research designs by providing larger sample sizes and higher real-world validity compared to randomized controlled trials [26]. However, for complex interventions, a purely quantitative evaluation that ignores process factors is considered insufficient [28]. As noted by several interviewees, Skivington et al. [28] advocate for incorporating process evaluation along with qualitative and mixed methods to improve data quality and provide insights beyond mere effectiveness. Furthermore, participatory and transdisciplinary research that involves stakeholders from various disciplines and sectors, along with individuals with lived experience, relatives, and the local community, not only enhances the relevance and applicability of findings but also strengthens commitment to SP initiatives [35, 37–39]. In line with the research-implementation gap highlighted by some interviewees in our study, O’Connor and Portzky [35] noted that new developments in suicide research often fail to be translated into practice, highlighting a significant potential for improvement.

### Role of reliable suicide data and opportunities for improvement

#### National suicide statistics

Experts in our study rated the quality of national suicide statistics in Germany, Austria, and Switzerland as comparatively high. Nevertheless, they noted limitations regarding the reliability of these data, a lack of detailed information, and delays in publication. Suicide statistics are crucial for understanding the patterns and trends in suicidality within the population. They help identify risk factors, develop targeted prevention measures, evaluate broad SP interventions and strategies, raise awareness, and inform political decisions on resource allocation. For example, the analysis of national suicide statistics in the United States of America indicated that the release of the Netflix series “13 Reasons Why” (first season) in 2017 was associated with an increase in suicide rates among children and adolescents [40, 41]. The series portrays the story of an adolescent girl who commits suicide following a series of traumatic life events and, according to critics, did not adhere to media guidelines for responsible reporting on suicides [40, 42]. In this case, the analysis of national suicide statistics has made it possible to validate experts’ concerns, underscore the potential severe consequences of media failure, and reinforce the implementation of targeted SP measures [41].

However, several limitations cast doubt on the validity and reliability of suicide statistics. In line with experts’ statements in our study, prior research highlighted issues of suicide underreporting and misclassification. A systematic review on the reliability of national suicide statistics by Tøllefsen et al. [14] showed that 12 of 31 included studies identified suicide underreporting of more than 30%. Suicides may be incorrectly recorded due to inherent flaws in the procedures for classifying deaths [43]. Inaccuracies may stem from the ambiguity of some deaths by suicide, limitations in the system for collecting suicide data, varying practices in suicide recording, as well as cultural, religious, financial, and legal considerations [43–46]. Determining the cause of death can be difficult, for example, when distinguishing between suicide and accidental or natural death requires a subjective interpretation of the deceased’s intent [14]. As some experts in our study speculated, closer examination of ‘deaths of undetermined intent’, ‘accidents’, and ‘homicides’ may reveal additional suicides [14, 43, 47]. A thorough forensic and psychological autopsy is likely the most valid method for determining the cause of death [14, 48]. According to a systematic review by Shojania et al. [49], autopsies can reveal considerable inaccuracies in death certificates, identifying a median major diagnostic error rate of 23.5%. According to Kapusta et al. [48], the national autopsy rate is a major predictor for suicide rates. However, autopsy rates are decreasing in most developed countries, for example, due to lower rates of request and consent [50], adversely affecting the reliability of suicide statistics. In addition to scrutinizing alternative causes of death, less apparent suicide methods such as poisoning, falling, and drowning - where misclassification is more likely - should be monitored [43].

Differences in suicide classification and recording methods limit the comparability of national suicide data across countries [14, 16]. However, experts in our study highlighted that such differences also exist between different actors at the regional level, such as the police, healthcare facilities, and emergency medical services. This shortcoming emphasizes the need for establishing a uniform definition of suicide, standard documenting and reporting practices, and adequate training of professionals involved in suicide data collection [27, 45, 46]. In line with experts’ statements from our study, a report on the quality of suicide data in Switzerland indicated that Swiss mortality statistics lack important background information, such as details on the suicide method and location of the incidents [27]. Additionally, the report recommends accelerating the process from the occurrence of death to the publication of mortality data, to facilitate prevention efforts and the corresponding evaluation [27].

Differences in suicide patterns across countries and regions, alongside changes in suicide rates, characteristics, and methods over time, underscore the importance of improving the comprehensiveness, reliability, and timeliness of national suicide statistics [15]. We recommend a multifaceted approach that includes standardizing suicide data collection [45], advancing national and regional suicide monitoring systems through cross-sector collaboration and agreement on leadership and financing, training for those included in collecting suicide data, and thorough investigation of ambiguous causes of death. Improvements to national suicide statistics should be informed by regional good practice systems, such as those in Carinthia, Austria [51, 52] and Zurich, Switzerland [53], which provide more detailed and current data.

#### Data on suicide attempts

The lack of a system for monitoring suicide attempts is an important gap in Germany, Austria, Switzerland, and most other countries worldwide [16]. Monitoring suicide attempts is inherently more challenging than recording deaths by suicide, as they are even more likely to remain concealed. To address this issue, data collection on suicide attempts needs to be standardized among all stakeholders, and a consensus on a clear definition of a suicide attempt should be developed to ensure consistency and accuracy [16, 27]. Since some individuals are treated in a hospital after a suicide attempt, or attempt suicide during hospitalization, hospital data are a valuable source of information [27]. Ideally, however, data from multiple sources, including hospitals, mental health facilities, emergency services, police protocols, and coroners’ reports, should be integrated to capture as complete a depiction of suicide attempts as possible. Recording suicide attempts across different contexts requires close cross-sectoral collaboration, involving the health sector, criminal justice system, educational institutions, and social services. Conducting regular and representative surveys to collect self-reported data on suicide attempts and ideation can bridge significant data gaps. Additionally, establishing accessible community-based reporting systems that allow healthcare providers, educators, and community members to confidentially report suicide attempts could be a valuable complementary measure [15].

In 2016, the World Health Organization published a ‘Practice manual for establishing and maintaining surveillance systems for suicide attempts and self-harm’ [16], which includes examples of national statistics and registries dedicated to documenting hospital-presented suicide attempts. Notable initiatives, such as the database implemented by the Werner Felber Institute in Germany for recording suicides and suicide attempts in hospitals across German-speaking countries [54], provide valuable good practice models for developing a national, centralized database that aggregates information from multiple sources.

### Limitations

This study details the opinions, experiences, and viewpoints of 36 individuals, which may not represent all SP experts in Germany, Austria, and Switzerland. The selection of experts was guided by the researchers’ discretion, suggesting that other experts might have emphasized different issues or opinions. Such variability, while common in qualitative research, limits the representativeness of our results. Furthermore, since the interviews were conducted exclusively with SP experts, there might be vested interests and a possible bias towards certain topics, such as resource allocation for suicide research. Our manuscript focuses on key findings that were supported by several participants. Therefore, we believe that the potential for bias stemming from unsubstantiated beliefs or emotional influences is minimal [17]. The focus on countries with similar socio-cultural and political-organizational characteristics (Germany, Austria, Switzerland) may limit the applicability of our findings to countries with different political systems. Additionally, conducting interviews primarily in German is a limitation, especially considering Switzerland’s multilingualism and federal structure. Consequently, the French- and Italian-speaking cantons are not adequately represented in our study [17].

## Conclusion

This study provides actionable recommendations and highlights existing good practice approaches to support decision-makers and provide guidance for advancing SP on a broader scale. SP evaluations could be improved by integrating both traditional and innovative research designs, including intermediate outcome criteria and factors concerning the implementation process alongside primary suicide endpoints, and engaging relevant stakeholders (e.g., individuals with lived experience, health service providers) through participatory and transdisciplinary research. To facilitate these efforts, it is important to prioritize evaluations and provide adequate financial support. Strong evidence for the effectiveness of SP interventions not only facilitates negotiations with potential funders but also improves support for individuals at suicide risk and helps counteract societal myths about the inevitability of suicide.

Despite the critical importance of reliable and timely suicide data, national statistics in Germany, Austria, and Switzerland reveal specific gaps and limitations. Adopting standardized data collection methods, enhancing cross-sector collaboration, ensuring timely data dissemination, and establishing national monitoring systems could significantly improve the quality of suicide data, thereby supporting the development and evaluation of targeted SP measures and strategies.

### Electronic supplementary material

Below is the link to the electronic supplementary material.

**Supplementary Material 1**: Interview guide.

## Data Availability

The dataset generated and analyzed during this study is not publicly available because it contains information that could compromise the privacy of the interviewees. Reasonable requests to access parts of the dataset can be directed to the corresponding author (SW, sophia.werdin@swisstph.ch).

## Abbreviations

COVID-19: Coronavirus disease 2019
SP: Suicide Prevention
