# Supplementary material for: Challenges in the evaluation of suicide prevention measures and quality of suicide data in Germany, Austria, and Switzerland: findings from qualitative expert interviews
Source: BMC Public Health. 2024 Aug 13;24:2209. doi: 10.1186/s12889-024-19726-w (PMC11323587; doi:10.1186/s12889-024-19726-w)
Supplement: Supplementary file 1 — Supplementary Material 1: Interview guide. [file 12889_2024_19726_MOESM1_ESM.docx]

**Additional file 1.** Interview guide (previously published in: *Werdin S, Wyss K. Advancing*

*suicide prevention in Germany, Austria and Switzerland: a qualitative study. Front Public Health. 2024;12:1378481. doi: 10.3389/fpubh.2024.1378481*)

**Introduction**

- Please describe your professional role in the area of suicide prevention.
- How long have you been professionally engaged in suicide prevention efforts?
- Approximately what percentage of your working time is dedicated to suicide prevention activities?

**Aspects on the national SP approach**

- In your opinion, what is the significance of suicide prevention in [*Country*]?
- Could you please briefly explain the approach taken in planning suicide prevention measures?
- To what extent are target groups involved in the planning of suicide prevention measures?
- How prevalent are suicide prevention measures in rural areas?
- How do you assess the collaboration among various sectors and stakeholders in suicide prevention? (e.g., media, health care, education, NGOs)
- How do you assess the collaboration between different treatment settings in suicide prevention? (e.g., inpatient and outpatient services)

**Evaluation of suicide prevention measures**

- How do you evaluate the activities related to the evaluation of individual suicide prevention measures?
- To what extent is the overall suicide prevention strategy in [*Country*] evaluated?
- Who is typically responsible for the evaluation of the strategy and individual measures?

**Effectiveness of suicide prevention measures**

- In your opinion, which elements of a suicide prevention strategy are most effective?
- What factors influence the acceptance and utilization of suicide prevention services and projects?

**Availability and quality of suicide data and research in suicide prevention**

- How do you assess the availability and quality of epidemiological data on suicides and suicide attempts in [*Country*]?
- How do you assess the available evidence in the field of suicide prevention?
- To what extent are the ongoing suicide prevention activities based on scientific findings?
- Which aspects are you investigating in your (suicide prevention) research?
- What scientific methods do you employ in your research?
- Which areas should future research projects in suicide prevention focus on?

**Challenges in suicide prevention**

- What challenges and problems do you encounter in your work in suicide prevention?

**Impact of the coronavirus disease 2019 (COVID-19) pandemic on suicide prevention**

- To what extent has the COVID-19 pandemic impacted the need for suicide prevention measures?
- How has the COVID-19 pandemic influenced your research activities in this field?
- How have suicide prevention measures needed to be adapted in the COVID-19 pandemic?

**Best practice elements and optimization potentials**

- What measures or aspects of suicide prevention are particularly well implemented in [*Country*]?
- How are 'Best Practices' in suicide prevention communicated and transferred? (also internationally)
- Where do you see potential for optimization in the field of suicide prevention in [*Country*]?

**Final thoughts**

- Is there anything else you would like to add or emphasize regarding the topic of suicide prevention?
